# Assessment and analysis of patient safety competency of Chinese nurses with associate degrees: A cross‐sectional study

**DOI:** 10.1002/nop2.640

**Published:** 2020-10-06

**Authors:** Lupei Yan, Lili Yao, Yuerong Li, Hao Chen

**Affiliations:** ^1^ Department of Anesthesiology The First Affiliated Hospital of Chongqing Medical University Chongqing China; ^2^ Department of Epidemiology School of Public Health and Management Chongqing Medical University Chongqing China

**Keywords:** China, nurses with associate degrees, nursing education, patient safety competency, quality and safety

## Abstract

**Aim:**

To analyse the patient safety competency (PSC) of Chinese nurses with associate degrees (ADNs) and explore factors.

**Design:**

A cross‐sectional study.

**Methods:**

A convenience sample of 451 ADNs working in 18 hospitals located in Chongqing city of China was investigated using the Patient Safety Competency Nurse Evaluation Scale (PSCNES). Descriptive and inferential statistics were used to analyse the data.

**Results:**

ADNs had a moderate level of PSC. In terms of the six dimensions of PSC, ADNs performed well in clinical practice and safety risk management, while they performed poorly in patient‐centred care and patient safety culture. Statistically significant differences were reported in two items. Firstly, ADNs who have participated in patient safety training had a higher level in all dimensions of PSC than those who have not participated in related training. Secondly, ADNs without professional titles had a higher level of patient safety culture than those with professional titles.

## 1 INTRODUCTION

In 2000, the Institute of Medicine (IOM) published a landmark report *To Err Is Human: Building a Safer Health System*, which revealed at least 44,000 and probably up to 98,000 inpatients died in hospitals every year due to medical errors, whereas 58% of medical errors were preventable (Institute of Medicine, 2000). IOM defined such preventable medical error as “patient safety” (Institute of Medicine, 2004). The publication of this report has aroused worldwide attention and consideration of patient safety issues. In May 2019, the 72nd World Health Assemble held in Switzerland endorsed the establishment of World Patient Safety Day to be observed annually on 17 September. Subsequently, the National Health Commission of the People's Republic of China actively responded to the initiative and encouraged healthcare workers to engage in patient safety.

Patient safety is the core indicator of quality of the healthcare industry. However, with people's increasing awareness of the frequent occurrence of preventable medical errors in health care, patient safety issues have been recognized as a pressing challenge. In the high‐risk healthcare industry, clinical nurses, the largest group of health professionals, have the ethical and moral responsibility to provide high‐quality safe practice (Earle‐Foley et al., 2012). Accordingly, it is essential to accurately assess nurses’ patient safety competency (PSC). In China, nurses with associate degrees (ADNs) are in a prominent position to guarantee patients’ safety, because they are the largest nursing team in the health workforce. This paper assessed ADNs’ PSC and identified which weaknesses should be strengthened through future patient safety education. More knowledge about this will help policymakers and educators to shape correlative programmes and policies.

### 1.1 Background

Patient safety is a health issue of global interest, and nurses should be competent in their ability to provide care services based on relevant nursing standards. Nursing competency consists of core abilities required for performing one's role as a nurse (Fukada, 2018), and PSC refers to the knowledge, attitudes and skills that nurses should possess to prevent patients from the injuries of medical errors (Jang & Lee, 2017). Besides, proper tests can be conducive to assessing nurses’ PSC, as diagnostic tools for identifying strengths and weaknesses, to guide competency improvements and for benchmarking. However, a pervading challenge in developing instruments specifically to assess nurses’ PSC is the lack of clarity about applicable patient safety structures (Levett‐Jones et al., 2020). To address this issue, Levett‐Jones designed and validated the Patient Safety Competency Framework (PSCF) by a modified Delphi technique (Levett‐Jones et al., 2017). The PSCF includes skill and knowledge statements categorized into nine domains (nine core competencies): therapeutic communication, person‐centred care, teamwork and collaborative practice, preventing, minimizing and responding to adverse events, cultural competency, infection prevention and control, medication safety, evidence‐based practice and clinical reasoning. The framework was structured with reference to Miller's pyramid of competency. In the PSCF, firstly, knowledge is conceptualized as the foundation for competency. Next, nurses must know how to apply knowledge using cognitive skills such as critical thinking. Finally, nurses do with their knowledge and skills in clinical practice.

Based on PSCF, Jones designed a quiz to measure final year nursing students’ knowledge about pivotal patient safety concepts. Moreover, we find most existing studies focused on nursing students’ PSC (Han et al., 2020). Lee used the Patient Safety Competency Self‐Evaluation (PSCSE) tool designed by his team to measure Korean nursing students’ PSC, including knowledge, attitudes and skills (Lee et al., 2016). Alquwez used the Health Professional Education in Patient Safety Survey (H‐PEPSS) to collect data on the perceived PSC of Saudi baccalaureate nursing students. The H‐PEPSS has six areas: communicating effectively, working in teams with other health professionals, understanding human, recognizing and responding to adverse events, managing safety risks and environmental factors that influence patient safety and culture of safety (Alquwez et al., 2019). However, few studies described the PSC of ADNs and exactly what they should learn. In China, the Associate degree in Nursing programme is a three‐year programme (with a 2‐year classroom and 1‐year clinical courses). Compared with baccalaureate nurses (a 4‐year programme), ADNs are a high‐risk group for the occurrence of nursing defects due to the lack of solid professional knowledge, the weakness of safe consciousness and inadequate understanding of medical errors (Lee et al., 2016). Therefore, it is vital to evaluate ADNs’ PSC and this research gap is addressed by the current study.

A few studies have examined the factors bound up with nursing students’ PSC: gender and years of study (Alquwez et al., 2019), clinical learning environments (Bianchi et al., 2016), clinical career (Jin & Yi, 2019), educational background (Anbari et al., 2019; Lee et al., 2016) and patient safety courses (Kim et al., 2019). These results make a contribution to providing productive insights and reference for strengthening participants’ PSC. Accordingly, this study examined factors affecting ADNs’ PSC to provide valuable insights for medical managers and nurse educators to implement patient safety education reform and enhance ADNs’ PSC.

The aim of this paper was to describe the PSC of Chinese ADNs and explore factors. The research questions were as follows:

1. What is the level of PSC of Chinese ADNs and what are their strengths and weaknesses.
2. What factors affect the level of PSC of Chinese ADNs.

## 2 METHODS

### 2.1 Study design

The study adhered to the STROBE guideline for cross‐sectional studies. This was a descriptive, cross‐sectional study to evaluate the PSC of ADNs who were participating in an ADN‐to‐BSN curriculum of a university in Chongqing, China, and to identify related influence factors. This ADN‐to‐BSN curriculum was held on weekends, which offered ADNs the opportunity to complete their BSN degrees after work.

### 2.2 Participants

Four hundred and fifty one ADNs from 18 hospitals located in Chongqing, China, participated in this study. All ADNs were licensed Registered Nurses (RNs) Certification in China and got associate degrees. The inclusion criteria for this study were as follows: (a) ADNs; (b) RNs; (c) with Chinese nationality; and (d) voluntary participation.

### 2.3 Instrument

We used a questionnaire in this study. The first part of the questionnaire collected demographic data (gender, age, marital status, working years, professional titles, working posts, hospitals, clinical settings, whether you have participated in patient safety training and whether you have participated in teamwork training). Professional titles can be regarded as the ratings of nurses. Nurses with different levels of nursing skills and working experience have different professional titles, including primary, intermediate and senior. Working posts are the same as clinical roles, such as head nurses and nurse educators.

In the second part, we used the Patient Safety Competency Nurse Evaluation Scale (PSCNES) developed by Wang (2018). The PSCNES is comprised of 35 items divided into dimensions of patient‐centred care (4 items), safety risk management (10 items), evidence‐based nursing practice (5 items), patient safety culture (4 items), clinical practice (5 items) and continuous quality improvement (7 items). Participants evaluate whether their abilities are consistent with the item statements and use a 5‐point Likert scale to score (1 = strongly inconsistent–5 = strongly consistent). The scores for each dimension are calculated to obtain an overall score, and the overall score ranges from 0–175. A high score signifies the better level of PSC. PSCNES’s Cronbach's alpha ranged from 0.76–0.91 for the six dimensions, which showed good internal consistency reliability (Wang, 2018). And the computed KMO value was 0.970, Bartlett χ^2^ = 12,112.30 (*p* < .01), which showed acceptable construct validity. The scale was used in this study after obtaining permission from the copyright holder via email.

### 2.4 Data collection

Data were collected from October–December 2019. The first author visited a university in Chongqing, China, and provided information about the research (i.e. significance of the study, risks and benefits of participation) for eligible ADNs during the break. ADNs who chose to participate were asked to sign an informed consent, then complete the questionnaire and submit it to the first author. Participants were asked not to indicate their names in the survey. 457 questionnaires were distributed and collected, including 451 valid questionnaires, after six invalid questionnaires with missing or similar items were eliminated, and the response rate for valid questionnaires was 98.7%. This study was approved by the ethics committee (no. 2020‐76).

### 2.5 Data analysis

All statistical analyses were performed with SPSS version 22.0. Descriptive statistics (i.e. percentage, frequency count) were calculated for the demographic profiles of participants. Means and standard deviations were calculated for ADNs’ PSC. The *t* test and one‐way analyses of variance were computed to examine the association between PSC and demographics. In addition, a multiple stepwise regression analysis was used to determine the factors affecting the ADNs' PSC. The level of significance was set at .05.

## 3 RESULTS

### 3.1 Demographics and scores of PSCNES

There were 451 ADNs enrolling in this study. Table 1 reflects the participants' demographic characteristics and differences in scores of PSCNES. Most of respondents were the young (20–30 years old, 94.68%) and female (98.45%). Many respondents had less than five years of working experience (84.04%), so they hardly had professional titles (22.39%) and working posts (12.86%). Almost half the respondents reported they have participated in patient safety training (51.66%) and teamwork training (44.57%), respectively.

**TABLE 1:** Characteristics and analysis of scores of PSCNES (*x* ± *s*, *N* = 451)

Items	*N* (%)	Scores of PSCNES						
		Total	Patient‐centred care	Safety risk management	Evidence‐based nursing practice	Patient safety culture	Clinical practice	Continuous quality improvement
Sex								
Male	7 (1.55)	136.00 ± 11.78	14.57 ± 1.51	38.47 ± 2.99	19.43 ± 2.37	15.14 ± 2.34	20.43 ± 1.13	27.86 ± 2.73
Female	444 (98.45)	137.20 ± 16.87	15.20 ± 2.26	40.18 ± 4.83	19.61 ± 2.76	14.58 ± 2.80	20.25 ± 2.66	27.39 ± 3.93
*t* (*p* value)		−0.187 (.852)	−0.735 (.463)	−0.876 (.381)	−0.171 (.864)	0.532 (.595)	0.177 (.859)	0.316 (.752)
Working years								
≤5	379 (84.04)	137.67 ± 17.28	15.26 ± 2.31	40.19 ± 4.94	19.72 ± 2.81	14.70 ± 2.85	20.31 ± 2.72	27.50 ± 3.94
>5	72 (15.96)	134.61 ± 13.81	14.85 ± 1.92	39.96 ± 4.03	19.03 ± 2.39	13.99 ± 2.39	19.97 ± 2.20	26.82 ± 3.76
*t* (*p* value)		1.417 (.157)	1.421 (.156)	0.426 (.671)	1.952 (.052)	1.994 (.047)*	1.134 (.259)	1.355 (.176)
Professional titles								
No	350 (77.61)	137.97 ± 17.07	15.30 ± 2.29	40.22 ± 4.88	19.73 ± 2.80	14.78 ± 2.80	20.35 ± 2.69	27.59 ± 3.90
Yes	101 (22.39)	134.46 ± 15.57	14.82 ± 2.09	39.90 ± 4.54	19.16 ± 253	13.92 ± 2.67	19.93 ± 2.45	26.72 ± 3.93
*t* (*p* value)		1.855 (.064)	1.883 (.060)	0.593 (.554)	1.860 (.063)	2.736 (.006)*	1.393 (.164)	1.956 (.051)
Working posts								
No	393 (87.14)	137.75 ± 17.13	15.28 ± 2.26	40.32 ± 4.85	19.71 ± 2.81	14.66 ± 2.85	20.31 ± 2.67	27.47 ± 3.98
Yes	58 (12.86)	133.33 ± 13.83	14.59 ± 2.13	38.98 ± 4.36	18.91 ± 2.17	14.07 ± 2.32	19.90 ± 2.45	26.88 ± 3.45
*t* (*p* value)		1.876 (.061)	2.204 (.028)*	1.989 (.047)*	2.061 (.040)*	1.757 (.082)	1.101 (.272)	1.069 (.286)
Marital Status								
Unmarried	338 (74.94)	137.17 ± 17.16	15.23 ± 2.31	40.09 ± 4.48	19.60 ± 2.79	14.67 ± 2.77	20.21 ± 2.68	27.36 ± 3.93
Married	113 (25.06)	137.21 ± 15.79	15.09 ± 2.11	40.32 ± 4.59	19.61 ± 2.66	14.32 ± 2.85	20.39 ± 2.54	27.47 ± 3.92
*t* (*p* value)		−0.022 (.983)	0.562 (.574)	−0.432 (.666)	−0.012 (.990)	1.140 (.255)	−0.644 (.520)	−0.255 (.799)
Patient safety training								
No	218 (48.34)	132.42 ± 15.13	14.55 ± 2.02	39.12 ± 4.40	18.91 ± 2.53	13.78 ± 2.71	19.74 ± 2.53	26.33 ± 3.78
Yes	233 (51.66)	141.63 ± 17.08	15.80 ± 2.30	41.11 ± 4.98	20.25 ± 2.79	15.34 ± 2.65	20.73 ± 2.66	28.39 ± 3.79
*t* (*p* value)		−6.044 (<.001)*	−6.129 (<.001)*	−4.500 (<.001)*	−5.332 (<.001)*	−6.206 (<.001)*	−4.069 (<.001)*	−5.793 (<.001)*
Teamwork training								
No	250 (55.43)	133.84 ± 16.18	14.71 ± 2.14	39.50 ± 4.65	19.09 ± 2.67	13.95 ± 2.81	19.93 ± 2.65	26.66 ± 3.91
Yes	201 (44.57)	141.34 ± 16.65	15.79 ± 2.25	40.96 ± 4.88	20.20 ± 2.72	15.38 ± 2.56	20.66 ± 2.58	28.31 ± 3.73
*t* (*p* value)		−4.831 (<.001)*	−5.195 (<.001)*	−3.24 (<.001)*	−4.519 (<.001)*	−5.587 (<.001)*	−2.938 (.003)*	−4.549 (<.001)*
Age								
≤20	8 (1.77)	141.25 ± 14.73	16.00 ± 2.45	41.00 ± 3.82	19.88 ± 2.70	15.00 ± 2.67	20.75 ± 2.05	28.63 ± 2.92
21–30	427 (94.68)	137.22 ± 16.73	15.20 ± 2.26	40.16 ± 4.78	19.60 ± 2.75	14.59 ± 2.79	20.26 ± 2.63	27.42 ± 3.88
>30	16 (3.55)	133.94 ± 19.85	14.69 ± 2.06	39.50 ± 5.93	19.56 ± 2.92	14.31 ± 3.03	19.81 ± 3.15	26.06 ± 5.07
*F* (*p* value)		0.533 (.587)	0.915 (.401)	0.271 (.762)	0.041 (.960)	0.164 (.849)	0.365 (.695)	1.330 (.266)
Clinical settings								
General intern	151 (33.48)	138.70 ± 17.32	15.20 ± 2.39	40.58 ± 4.95	19.76 ± 2.78	14.94 ± 2.72	20.26 ± 2.81	27.95 ± 4.02
General surgery	104 (23.06)	139.17 ± 16.44	15.37 ± 2.25	40.79 ± 4.80	20.03 ± 2.77	14.71 ± 2.87	20.61 ± 2.38	27.67 ± 3.74
Others	196 (43.46)	134.95 ± 16.39	15.10 ± 2.15	31.48 ± 4.63	19.27 ± 2.68	14.24 ± 2.78	20.06 ± 2.63	26.81 ± 3.87
*F* (*p* value)		3.097 (.046)*	0.481 (.618)	3.475 (.032)*	2.986 (.051)	2.809 (.061)	1.478 (.229)	4.027 (.018)

* *p* < .05.

As indicated in Table 2, the average overall score for PSCNES was 137.18 (*SD* 16.80), the scoring rate was 78.39% (137.18/175), and the mean of a single item was 3.92 (*SD* 0.48). In terms of six dimensions of PSCNES, the dimension “clinical practice” received the highest mean (4.05 *SD* 0.53), followed by “safety risk management” (4.02 *SD* 0.48), “evidence‐based nursing practice” (3.92 *SD* 0.55), “continuous quality improvement” (3.91 *SD* 0.56) and “patient‐centred care” (3.80 *SD* 0.56). The dimension “patient safety culture” received the lowest mean (3.65 *SD* 0.70).

**TABLE 2:** Scores of six dimensions of PSCNES (x ± s, *N* = 451)

Dimensions	Numbers of items	Minimum	Maximum	Average scores	Average scores of each item
Patient‐centred care	4	8	20	15.19 ± 2.25	3.80 ± 0.56
Safety risk management	10	26	50	40.15 ± 4.81	4.02 ± 0.48
Evidence‐based nursing practice	5	13	25	19.61 ± 2.75	3.92 ± 0.55
Patient safety culture	4	7	20	14.59 ± 2.79	3.65 ± 0.70
Clinical practice	5	11	25	20.25 ± 2.64	4.05 ± 0.53
Continuous quality improvement	7	15	35	27.39 ± 3.92	3.91 ± 0.56
Total score	35	91	175	137.18 ± 16.80	3.92 ± 0.48

### 3.2 Factors affecting the scores of PSCNES

The researchers conducted a multiple stepwise regression analysis to examine the factors affecting the scores of PSCNES (Table 3). Statistical significance of the variables of scores of PSCNES (whether you have participated in patient safety training, whether you have participated in teamwork training, working years, professional titles, working posts and clinical settings) was entered into the regression equation.

**TABLE 3:** Factors affecting the scores of PSCNES (*N* = 451)

Model	Variables	*B*	*SE*	Beta	*t*	*p*	*R* ^2^	Adjusted *R* ^2^
Model 1 (Overall scores)	(Constant)	132.422	1.095		120.923	<.001	.075	.073
	Patient safety training	9.209	1.524	0.274	6.044	<.001		
Model 2 (Patient‐centred care)	(Constant)	14.546	0.147		99.035	<.001	.077	.075
	Patient safety training	1.252	0.204	0.278	6.129	<.001		
Model 3 (Safety risk management)	(Constant)	39.124	0.319		122.730	<.001	.043	.041
	Patient safety training	1.988	0.444	0.207	4.482	<.001		
Model 4 (Evidence‐based nursing practice)	(Constant)	18.913	0.181		104.676	<.001	.060	.057
	Patient safety training	1.340	0.251	0.244	5.332	<.001		
Model 5 (patient safety culture)	(Constant)	13.965	0.194		71.912	<.001	.093	.089
	Patient safety training	1.546	0.251	0.277	6.154	<.001		
	Professional titles	−0.794	0.301	−0.119	−2.636	.009		
Model 6 (clinical practice)	(Constant)	19.739	0.176		112.269	<.001	.036	.033
	Patient safety training	0.995	0.245	0.189	4.069	<.001		
Model 7 (continuous quality improvement)	(Constant)	26.326	0.256		102.750	<.001	.070	.067
	Patient safety training	2.065	0.356	0.264	5.793	<.001		

In the seven multivariate regression models, the Durbin‐Watson test statistic result ranged from 1.965–2.129, which is close to 2, showing no autocorrelation. The tolerance was 0.999 to 1.000, more than 0.1, and the VIF was 1.000–1.001, 10 or less, showing no multicollinearity. The stepwise regression model indicated that patient safety training's participation affected the overall scores and the score of every dimension of PSCNES (*p* < .001). Besides, professional titles were related to the dimension “patient safety culture” (*p* = .009).

## 4 DISCUSSION

Nurses’ PSC plays a pivotal role in ensuring patient safety. To the best of our knowledge, this is the first study to examine ADNs’ PSC, and six domains of PSC—clinical practice, safety risk management, evidence‐based nursing practice, continuous quality improvement, patient‐centred care and patient safety culture. This study also revealed the factors affecting nurses’ PSC with demographics of respondents.

In our ADNs sample, the level of overall PSC was found to be moderate. This finding is consistent with previous research conducted among Korean nurses (Cho & Choi, 2018). When examining six fields of PSC separately, the dimensions “clinical practice” (4.05 *SD* 0.53) and “safety risk management” (4.02 *SD* 0.48) scored higher than the other four domains. These two dimensions can be attributed to safety practice skills. However, in a Korean study examining safety attitudes, knowledge and skills, the results showed that nurses were less skilful in the safety practice (Cho & Choi, 2018). Although the difference in conclusions is probable due to the adoption of different sampling variation and instruments, the results indicate that more studies should adopt the transcultural translations of the reliable instrument to assess all domains of domestic practitioners’ PSC, which can provide comparable baseline data for educators to formulate practical patient safety documents to strengthen nurses’ PSC.

Results showed that ADNs’ rates in the dimensions “evidence‐based nursing practice” (3.92 *SD* 0.55) and “continuous quality improvement” (3.91 *SD* 0.56) were similar to the mean of a single item of PSC (3.92 *SD* 0.48). Evidence‐based nursing practice refers to a conscious nursing thinking and application of various information sources, including the adoption of published literature in conjunction with clinical expertise and patient preferences and values (Horntvedt et al., 2018). And the continuous quality improvement is the adoption of reliable data and improvement methods (such as SBAR handoff) to learn which interventions, in which context, can comprehensively optimize care processes in healthcare systems (Gallen et al., 2019). Nurses are expected to generate evidence from clinical practice to establish guidelines for the continuous quality improvement. Furthermore, IOM also required all healthcare professionals to possess core competencies in quality and safe care. Two of the core competencies were evidence‐based practice and quality improvement (Balakas & Smith, 2016). Accordingly, it is crucial for nurses to master these two competencies to ensure patient safety.

The American Association of Colleges of Nursing (AACN) developed curricular criteria for baccalaureate, master's and Doctor of Nursing Practice projects to reinforce participants’ evidence‐based nursing competencies (American Association of Colleges of Nursing, 2008). And the Robert Wood Johnson Foundation also established a national educational programme for the nursing faculty to cultivate quality and safety competencies among students (Balakas & Smith, 2016). Nevertheless, relevant educational programmes, especially aimed at ADNs, were inadequate. Compared with baccalaureate nurses, ADNs had a weaker perception of improving their practice competencies (Loversidge et al., 2018), which led that they found it difficult to actively learn how to appraise evidence and engage in evidence‐based nursing and quality improvement implementation and collaboration. Therefore, to let ADNs adapt to the changing demands of health care, medical organizations should incorporate evidence‐based nursing and quality improvement into educational programmes from ADNs’ perspective. And these individual programmes would also be conducive to guiding ADNs to identify clinical concerns, evaluate and synthesize evidence to continuously improve the quality of safe care.

Our present results showed that the domains “patient safety culture” (3.65 *SD* 0.70) and “patient‐centred care” (3.80 *SD* 0.56) scored lower than the mean of a single item of PSC (3.92 *SD* 0.48), which may indicate that ADNs had a weakness in these two competencies comparing with the other four competencies of PSC.

Patient safety culture refers to the perceptions, attitudes, beliefs and values of patient safety shared among members of the healthcare organization (Zhong et al., 2019). Establishing a positive patient safety culture among clinical and administrative staff is essential for the improvement of safety quality. Our study showed that ADNs without professional titles had a higher level of patient safety culture than those with professional titles, and this result is inconsistent with many Chinese research (Wu et al., 2017). In China, nurses must have a certain number of working years and scientific research results and pass many examinations to obtain different levels of professional titles. To some extent, the professional title is a sign that reflects nurses’ technical level and working ability. Compared with ADNs without professional titles, ADNs with professional titles are likely to have a strong awareness of perceiving the risk of adverse events and a great patient safety culture. However, our result seems to be debatable. Given the measurement tool we used in this study is not an independent tool for measuring patient safety culture, which may lead to detection bias. Therefore, we suggest that future research can use specialized tools for patient safety culture, such as the Hospital Survey on Patient Safety Culture (Giai et al., 2017), to measure ADNs’ patient safety culture, in order to obtain more reliable data.

Patient‐centred care is a hot term in contemporary health care, referring to respectful care that includes attention to the patient's unique personalities, beliefs, circumstances, values and preferences in guiding shared clinical decision‐making (Mahoney et al., 2017). Successful patient‐centred care is both the patient and the nurse mutually agree to healthcare needs, treatments and experiences. Our results indicated that ADNs had a weakness in patient‐centred care competency, which is in accordance with Hwang's study (2019). According to Hwang, the degree of patient participation in patient safety was positively correlated with nurses’ patient‐centred care competency. However, the degree of patient engagement in clinical practice was not high and most patients preferred a passive involvement in safe care encountered and were accustomed to complying with medical instructions and responding to medical staff's questions (Hwang et al., 2019). Also, ADNs may lack the awareness of involving patients as care partners owing to busy work situations. In 2019, the China Hospital Association released the annual 10 patient safety goals. One of the 10 safety goals was that patient engagement in patient safety. Patient involvement in safe care is also suggested as a core element of patient‐centred care (Kitson et al., 2013). Thus, nursing leaders can enhance ADNs’ competency for patient‐centred care by promoting patient engagement in safety. The specific measures consist of supporting and reinforcing nurses’ skills in evaluating barriers to patient engagement, providing access to useful resources, empowering patients and encouraging patients to partner with ADNs.

Strong evidence from our study showed that ADNs who have participated in patient safety training had a higher level in all dimensions of PSC than those who have not participated in related training. This finding confirms the conclusion in other contemporary research that patient safety education has a positive effect on nurses’ PSC (Abdrbo, 2015; Hwang et al., 2016; VanDenKerkhof et al., 2017). According to Walpola et al. (2018), education has been widely regarded as a basic method to enhance patient safety in healthcare settings. Furthermore, Stefan, the president of the World Federation for Medical Education, ever said: “patient safety is a necessary competency and thus required to be introduced early and then strengthened throughout school education and continuing professional advancement” (Wu & Busch, 2019). Nevertheless, in this study, almost half the ADNs (48.34%) reported that they had never participated in patient safety training. And even ADNs who have participated in patient safety training, when examining the content and frequency of the training, mostly reported that they have only attended a safety lecture rather than systematic patient safety training. This result shows that patient safety education in China is still in its infancy and a systematic patient safety training system should be developed.

In 2009, the World Health Organization (WHO) developed the Patient Safety Curriculum Guide for Medical Schools based on the Australian National Patient Safety Education Framework (Nie et al., 2011). Since then, many countries have spent substantial resources and funds to implement patient safety education programmes and guarantee the quality of patient care (Alquwez et al., 2019). But different educational systems in different countries provide varying academic knowledge, professional experience, teaching styles, health equipment resources and financial supports (Alquwez et al., 2019). These factors may affect the performance of nurses’ PSC. Therefore, we suggest that hospitals, based on popularizing patient safety education as much as possible, restructure the patient safety education framework considering varied safe levels and pedagogical needs of nurses. In addition, to maximize the effect of patient safety education, healthcare policymakers should consider the differences in the medical systems and cultural backgrounds of different countries, fully draw on the experience of other countries and explore an optimal patient safety education programme suitable for their domestic conditions.

### 4.1 Limitations

Some limitations of our study should be acknowledged and considered. Firstly, the potential selection and response bias might arise from using a convenience sample and a self‐reported questionnaire. Additionally, generalization of results requires caution because the respondents (ADNs) were not representative of all nurses in China. Consequently, further work may be needed to definitely examine that the PSC of nurses with different academic degrees. And related clinical safety outcome index should be taken into consideration when appraising nurses’ PSC, such as incidence of complications, length of stay and patient satisfaction.

## 5 CONCLUSIONS

This study determined the PSC of Chinese ADNs from Chongqing city of 18 hospitals, analysed the scores of six dimensions of PSC and explored factors associated with PSC. Chinese ADNs are at a moderate level of PSC. In terms of the six domains of PSC, they perform better in clinical practice and safety risk management, while they have a weakness in patient‐centred care and patient safety culture. In addition, ADNs who have participated in patient safety training have a higher level in all areas of PSC and ADNs without professional titles perform well in patient safety culture. These findings provide valuable references aimed at improving the quality of safety care. For instance, nurse managers should adopt reliable instruments to regularly assess nurses’ PSC, which can guide the creation of safety policies and pedagogical interventions. Secondly, there is a need to develop a specialized curriculum to strengthen ADNs’ evidence‐based nursing practice and continuous quality improvement competencies. And nurses should learn to encourage patients to participate in patient safety. Moreover, it is necessary for hospital executives to popularize patient safety education as much as possible and form an individualized patient safety curriculum based on the actual needs of health care to ensure the continuous advancement of front‐line nurses’ PSC.

## CONFLICT OF INTEREST

The authors declare no conflict of interest.

## Authorship statement

All listed authors meet the authorship criteria, and all authors are in agreement with the content of the manuscript.

## Author contribution

All listed authors certified their contribution. Lupei Yan, Lili Yao and Yuerong Li: Study design. Lupei Yan and Lili Yao: Data collection. Lupei Yan and Hao Chen Data analysis. Lupei Yan and Yuerong Li: Manuscript writing and revisions for important intellectual content.

## Data Availability

The data sets used or analysed during the current study are available from the corresponding author on reasonable request.

## References

[nop2640-bib-0001] Abdrbo, A. A. (2015). Nursing informatics competencies among nursing students and their relationship to patient safety competencies: Knowledge, attitude and skills. Computers, Informatics, Nursing, 33(11), 509–514. 10.1097/CIN.0000000000000197 26524185

[nop2640-bib-0002] Alquwez, N. , Cruz, J. P. , Alshammari, F. , Felemban, E. M. , Almazan, J. U. , Tumala, R. B. , Alabdulaziz, H. M. , Alsolami, F. , Silang, J. , & Tork, H. (2019). A multi‐university assessment of patient safety competence during clinical training among baccalaureate nursing students: A cross‐sectional study. Journal of Clinical Nursing, 28(9–10), 1771–1781. 10.1111/jocn.14790 30667103

[nop2640-bib-0003] American Association of Colleges of Nursing. (Published 2008). The essentials of baccalaureate education for professional nursing practice. *Essentials Series* Retrieved from http://www.aacn.nche.edu/education-resources/BaccEssentials08.pdf

[nop2640-bib-0004] Anbari, A. B. , Vogelsmeier, A. , & Dougherty, D. S. (2019). Patient safety communication among differently educated nurses: Converging and diverging meaning systems. Western Journal of Nursing Research, 41(2), 171–190. 10.1177/0193945917747600 29243561

[nop2640-bib-0005] Balakas, K. , & Smith, J. R. (2016). Evidence‐based practice and quality improvement in nursing education. The Journal of Perinatal & Neonatal Nursing, 30(3), 191–194. 10.1097/JPN.0000000000000197 27465447

[nop2640-bib-0006] Bianchi, M. , Bressan, V. , Cadorin, L. , Pagnucci, N. , Tolotti, A. , Valcarenghi, D. , Watson, R. , Bagnasco, A. , & Sasso, L. (2016). Patient safety competencies in undergraduate nursing students: A rapid evidence assessment. Journal of Advanced Nursing, 72(12), 2966–2979. 10.1111/jan.13033 27222204

[nop2640-bib-0007] Cho, S. M. , & Choi, J. (2018). Patient safety culture associated with patient safety competencies among registered nurses. Journal of Nursing Scholarship, 50(5), 549–557. 10.1111/jnu.12413 30009449

[nop2640-bib-0008] Earle‐Foley, V. , Myrick, F. , Luhanga, F. , & Yonge, O. (2012). Preceptorship: Using an ethical lens to reflect on the unsafe student. Journal of Professional Nursing, 28(1), 27–33. 10.1016/j.profnurs.2011.06.005 22261602

[nop2640-bib-0009] Fukada, M. (2018). Nursing competency: Definition, structure and development. Yonago Acta Medica, 61(1), 1–7. 10.33160/yam.2018.03.001 29599616 PMC5871720

[nop2640-bib-0010] Gallen, A. , Kodate, N. , & Casey, D. (2019). How do nurses and midwives perceive their preparedness for quality improvement and patient safety in practice? A cross‐sectional national study in Ireland. Nurse Education Today, 76, 125–130. 10.1016/j.nedt.2019.01.025 30784840

[nop2640-bib-0011] Giai, J. , Boussat, B. , Occelli, P. , Gandon, G. , Seigneurin, A. , Michel, P. , & François, P. (2017). Hospital survey on patient safety culture (HSOPS): Variability of scoring strategies. International Journal for Quality in Health Care, 29(5), 685–692. 10.1093/intqhc/mzx086 28992144

[nop2640-bib-0012] Han, Y. , Kim, J. S. , & Seo, Y. (2020). Cross‐sectional study on patient safety culture, patient safety competency and adverse events. Western Journal of Nursing Research, 42(1), 32–40. 10.1177/0193945919838990 30915918

[nop2640-bib-0013] Horntvedt, M. T. , Nordsteien, A. , Fermann, T. , & Severinsson, E. (2018). Strategies for teaching evidence‐based practice in nursing education: A thematic literature review. BMC Medical Education, 18(1), 172 10.1186/s12909-018-1278-z 30055612 PMC6064179

[nop2640-bib-0014] Hwang, J. I. , Kim, S. W. , & Chin, H. J. (2019). Patient participation in patient safety and its relationships with nurses' patient‐centered care competency, teamwork and safety climate. Asian Nursing Research, 13(2), 130–136. 10.1016/j.anr.2019.03.001 30898671

[nop2640-bib-0015] Hwang, J. I. , Yoon, T. Y. , Jin, H. J. , Park, Y. , Park, J. Y. , & Lee, B. J. (2016). Patient safety competence for final‐year health professional students: Perceptions of effectiveness of an interprofessional education course. Journal of Interprofessional Care, 30(6), 732–738. 10.1080/13561820.2016.1218446 27705029

[nop2640-bib-0016] Institute of Medicine. (2000). (US) Committee on Quality of Health Care in America In Kohn L. T., Corrigan J. M., & Donaldson M. S. (Eds.). To err is human: Building a safer health system. National Academies Press (US). 25077248

[nop2640-bib-0017] Institute of Medicine . (2004). (US) Committee on Data Standards for Patient Safety In Aspden P., Corrigan J. M., Wolcott J., & Erickson S. M. (Eds.). Patient safety: Achieving a new standard for care. National Academies Press (US). 25009854

[nop2640-bib-0018] Jang, H. , & Lee, N. J. (2017). Patient safety competency and educational needs of nursing educators in South Korea. PLoS One, 12(9), e0183536 10.1371/journal.pone.0183536 28873099 PMC5584796

[nop2640-bib-0019] Jin, J. , & Yi, Y. J. (2019). Patient safety competency and the new nursing care delivery model. Journal of Nursing Management, 27(6), 1167–1175. 10.1111/jonm.12788 31069860

[nop2640-bib-0020] Kim, Y. M. , Yoon, Y. S. , Hong, H. C. , & Min, A. (2019). Effects of a patient safety course using a flipped classroom approach among undergraduate nursing students: A quasi‐experimental study. Nurse Education Today, 79, 180–187. 10.1016/j.nedt.2019.05.033 31153088

[nop2640-bib-0021] Kitson, A. , Marshall, A. , Bassett, K. , & Zeitz, K. (2013). What are the core elements of patient‐centred care? A narrative review and synthesis of the literature from health policy, medicine and nursing. Journal of Advanced Nursing, 69(1), 4–15. 10.1111/j.1365-2648.2012.06064.x 22709336

[nop2640-bib-0022] Lee, N. J. , Jang, H. , & Park, S. Y. (2016). Patient safety education and baccalaureate nursing students' patient safety competency: A cross‐sectional study. Nursing & Health Sciences, 18(2), 163–171. 10.1111/nhs.12237 26306563

[nop2640-bib-0023] Levett‐Jones, T. , Andersen, P. , Bogossian, F. , Cooper, S. , Guinea, S. , Hopmans, R. , McKenna, L. , Pich, J. , Reid‐Searl, K. , & Seaton, P. (2020). A cross‐sectional survey of nursing students' patient safety knowledge. Nurse Education Today, 88, 104372– 10.1016/j.nedt.2020.104372. 32143174

[nop2640-bib-0024] Levett‐Jones, T. , Dwyer, T. , Reid‐Searl, K. , Heaton, L. , Flenady, T. , Applegarth, J. , Guinea, S. & Andersen, P. (2017). Patient safety competency framework (PSCF) for nursing students. Retrieved from http://psframework.wpengine.com/wp-content/uploads/2018/01/PSCF_Brochure_UTS-version_FA2-Screen.pdf

[nop2640-bib-0025] Loversidge, J. , Yen, P. Y. , Chipps, E. , Gallagher‐Ford, L. , Genter, L. , & Buck, J. (2018). Top‐of‐license nursing practice, part 2: Differentiating BSN and ADN perceptions of top‐of‐license activities. The Journal of Nursing Administration, 48(6), 329–334. 10.1097/NNA.0000000000000623 29794597

[nop2640-bib-0026] Mahoney, J. S. , Mulder, C. , Hardesty, S. , & Madan, A. (2017). Integrating caring into patient‐centered care through interprofessional education and ethics: The Caring Project. Bulletin of the Menninger Clinic, 81(3), 233–246. 10.1521/bumc_2017_81_02 28745943

[nop2640-bib-0027] Nie, Y. , Li, L. , Duan, Y. , Chen, P. , Barraclough, B. H. , Zhang, M. , & Li, J. (2011). Patient safety education for undergraduate medical students: A systematic review. BMC Medical Education, 11, 33 10.1186/1472-6920-11-33 21669007 PMC3128569

[nop2640-bib-0028] VanDenKerkhof, E. , Sears, N. , Edge, D. S. , Tregunno, D. , & Ginsburg, L. (2017). Patient safety in practical nurses' education: A cross‐sectional survey of newly registered practical nurses in Canada. Nurse Education Today, 51, 48–56. 10.1016/j.nedt.2017.01.003 28126688

[nop2640-bib-0029] Walpola, R. L. , McLachlan, A. J. , & Chen, T. F. (2018). A scoping review of peer‐led education in patient safety training. American Journal of Pharmaceutical Education, 82(2), 6110 10.5688/ajpe6110 29606704 PMC5869746

[nop2640-bib-0030] Wang, Q. (2018). Development of the patient safety competency nurse evaluation Scale. PhD thesis, Peking Union Medical College (In Chinese).

[nop2640-bib-0031] Wu, A. W. , & Busch, I. M. (2019). Patient safety: A new basic science for professional education. GMS Journal for Medical Education, 36(2), Doc21 10.3205/zma001229 30993179 PMC6446473

[nop2640-bib-0032] Wu, Y. J. , Yuan, Y. , Liu, A. M. , Zhu, L. X. , Wu, J. , & Li, B. G. (2017). The survey on perceptions of patient safety culture among nurses in tertiary hospitals in Kunming. Chinese Journal of Nursing, 52(6), 724–730. 10.3761/j.issn.0254-1769.2017.06.017

[nop2640-bib-0033] Zhong, X. , Song, Y. , Dennis, C. , Slovensky, D. J. , Wei, L. Y. , Chen, J. , & Ji, J. (2019). Patient safety culture in Peking University Cancer Hospital in China: Baseline assessment and comparative analysis for quality improvement. BMC Health Services Research, 19(1), 1008 10.1186/s12913-019-4837-z 31883512 PMC6935497

